# Modulation of gene expression in a human cell line caused by poliovirus, vaccinia virus and interferon

**DOI:** 10.1186/1743-422X-4-24

**Published:** 2007-03-05

**Authors:** Bjørn Grinde, Marc Gayorfar, Gunnar Hoddevik

**Affiliations:** 1 Division of Infectious Disease Control, Norwegian Institute of Public Health, PO Box 4404 Nydalen, 0403 Oslo, Norway

## Abstract

**Background:**

The project was initiated to describe the response of a human embryonic fibroblast cell line to the replication of two different viruses, and, more specifically, to look for candidate genes involved in viral defense. For this purpose, the cells were synchronously infected with poliovirus in the absence or presence of interferon-alpha, or with vaccinia virus, a virus that is not inhibited by interferon. By comparing the changes in transcriptosome due to these different challenges, it should be possible to suggest genes that might be involved in defense.

**Results:**

The viral titers were sufficient to yield productive infection in a majority of the cells. The cells were harvested in triplicate at various time-points, and the transcriptosome compared with mock infected cells using oligo-based, global 35 k microarrays. While there was very limited similarities in the response to the different viruses, a large proportion of the genes up-regulated by interferon-alpha were also up-regulated by poliovirus. Interferon-alpha inhibited poliovirus replication, but there were no signs of any interferons being induced by poliovirus. The observations suggest that the cells do launch an antiviral response to poliovirus in the absence of interferon. Analyses of the data led to a list of candidate antiviral genes. Functional information was limited, or absent, for most of the candidate genes.

**Conclusion:**

The data are relevant for our understanding of how the cells respond to poliovirus and vaccinia virus infection. More annotations, and more microarray studies with related viruses, are required in order to narrow the list of putative defence-related genes.

## Background

Microarrays offer an opportunity to investigate how viruses manipulate cells, and how the cells respond. Besides improving our understanding of cell-virus interactions, the method may lead to the discovery of novel cellular defense mechanisms.

Poliovirus belongs to the genus Enterovirus within the family *Picornaviridae*. Enteroviruses are highly prevalent in humans, and cause a variety of diseases. Although poliovirus infections are rare today, the strain employed in the present study (type 1 Sabin) is still used as a live vaccine, and may be considered a prototype enterovirus. Similarly, vaccinia virus is a typical pox virus. Enteroviruses are small RNA viruses, while vaccinia has a large DNA genome. Their biology is very different, but both replicate in the cytoplasm, and they can both be grown on human embryonic fibroblasts. We were interested in comparing their impact on these cells, measured as changes in the concentration of cellular transcripts.

Microarrays have been used to investigate the effect of a number of viruses on cellular transcripts. As to enteroviruses, there are one report on poliovirus [1] and a few on enterovirus 71 [2-4] using human cells, as well as two on coxsackievirus B3 in mice [5,6]. Vaccinia virus [7,8] and rabbitpox virus [9] have been examined using human cells, and variola virus in monkeys [10]. Although it is difficult to compare experiments based on different microarray platforms, these reports offered valuable information for the present analyses.

Type I interferons are widely known to help cells combat viral infections, primarily RNA viruses, but also DNA viruses [11,12]. There are three main versions of type I interferon (α, γ and ω), of which interferon-α is the more common, comprising a family of more than 20 genes. Microarrays have proven useful for investigating the joint effect of viruses and interferon [13]. In vivo, interferon-α is produced primarily by leukocytes, while fibroblasts are known to produce interferon-β [11]. Fibroblasts cells may, however, produce interferon-α upon priming. The microarray results should offer a clue as to whether any interferon genes were induced upon viral infection in the present cell line, and to what extent the cells responded to externally applied interferon-α.

Interferon-α (here referred to as Ifn) was included in the present experiments in order to help identify possible antiviral response genes. Vaccinia virus is resistant to interferon [14], and might be expected to elicit a different cellular response. Poliovirus was added both in the absence and presence of Ifn in order to demonstrate the effect of Ifn on viral replication, and to see whether the same set of genes would be induced by Ifn in the presence of virus. Poliovirus might be able to block the induction of some Ifn response genes [12].

Most of the previously published microarray reports used either clinical material or in vitro cell cultures that were not synchronously infected. Moreover, the arrays typically detected only subsets of the human transcriptosome. In the present effort, we pulse infected with doses sufficient to cause primary infection of a majority of the cells. Moreover, the cells were washed to remove unattached virus after 1 h of incubation, thus generating a synchronicity of viral replication. The microarrays employed contain 34 580 reporters (70-mer oligos from Operon), which presumably cover the predicted 25 000 human protein-expressing genes, and distinguish between a number of alternatively spliced mRNAs. The determination of global changes in gene expression caused by poliovirus, vaccinia virus or interferon, allowed for some interesting inferences as to the viral-host interaction.

## Results and Discussion

### Virus and interferon

HUGO gene symbols are used when available, otherwise the genes are referred to by their Operon Id. Complete data sets can be obtained from Gene Expression Omnibus under the accession no GSE5549.

Poliovirus is known to require some 7–10 h to produce novel viral particles, while vaccinia virus requires 6–8 h. The time points of 8 and 16 h were chosen for poliovirus. Initial experiments with 4 h (using cDNA arrays) suggested that at this time there were few changes in cellular RNA expression. As to vaccinia virus, however, there were ample changes after 4 h.

In the case of vaccinia virus, some 60–70% of the cells were infected by the single, pulse addition of virus at 2 MOI, as measured by immunofluorescense. Cytopathogenicity was observed after 2–3 days of culture, and the cells would eventually die. In the case of poliovirus the dose of virus was either 10 MOI (only virus) or 2 MOI (when evaluating the effect of Ifn). In both cases cytopathogenicity became apparent from approximately day 2, affecting some 70 – 90% of the cells. Thus a good majority of the cells were presumably infected at the initial round of viral addition. Depending somewhat on the dose, cells were dying between day 4 and day 7. The doses of Ifn and virus were calibrated to allow for a distinct effect of Ifn, but not total blockage of viral replication; thus Ifn offered sufficient protection to delay the start of cytopathogenicity to day 6 or 7. In the absence of viruses there were no signs of cellular decline.

### General effects on transcriptosome

When looking at all the data obtained from the oligo-based arrays, a total of 16572 different reporters gave signals above the cut-off in at least one experiment. These reporters corresponded to 10195 annotated genes. Many genes were represented by two or more reporters, thus the total number of genes expressed was estimated to be approximately 12000. In individual experiment (combining the three parallels) some 13 – 14000 reporters gave signals above cut-off. The lists of expressed reporters from different experiments were 80–90% overlapping. For simplicity, the discussion will refer to the reporter signals as reflecting the activity of genes.

Lists of up- or down-regulated genes were based on a log_2 _cut-off of, respectively, above 0.6 or below -0.6. The numbers of genes included in these lists for the various treatments of the cells are shown in Table 1. In the case of poliovirus, particularly at the early stage of viral infection, more genes were up-regulated than down-regulated. One dye-swap experiment was performed (using poliovirus infected and uninfected cells at 8 h), and in this case too there were more up-regulated genes, thus the difference was unlikely to reflect a bias due to labeling procedure. Moreover, the same was the case in comparable studies on other enteroviruses [3,5].

**Table 1:** Number of up- or down-regulated genes.

	Pol-8	Ifn-8	Pol+Ifn-8	Pol-16	Ifn-16	Pol+Ifn-16	Vac-4
Up-regulated	170	238	209	167	220	241	112
Down-regulated	11	29	124	123	126	52	205

The cells were incubated with poliovirus (Pol) and/or interferon-α (Ifn) for 8 or 16 h, and with vaccinia virus (Vac) for 4 h prior to microarray examination. The reported numbers are based on genes where the average change in three parallel experiments was more than 1.5 × (log_2 _ratio 0.6). The total number of expressed genes was 16572.

It may seem logical that effectors in general are more likely to turn on novel genes than to reduce the levels of mRNAs already present, particularly within the relatively short time frame of the present experiments. Somewhat surprisingly, in the case of vaccinia virus, more genes were down-regulated. This, however, was also the case in previously published experiments with the same virus, but different cells [7], suggesting that the data reflects a peculiarity of this virus.

Enteroviruses, including poliovirus, are known to inhibit host cell RNA synthesis by reducing the activity of all three host RNA polymerases [15]. It has been shown that ribosomal RNA synthesis is inhibited first, followed by mRNA synthesis, and finally tRNA, and that the inhibition is due to the degradation of host transcription factors utilized by the three RNA polymerases (see [16] and references therein). The yields of RNA extracted from poliovirus infected cells were on the average 40% lower than in control cells (compared to a 27% reduction in the Ifn treated cells), suggesting that the inhibition of RNA synthesis had started. However, when labeling for hybridization, the same amount of total RNA was used, thus the inhibition of RNA synthesis should only be noticeable on the arrays if there was an appreciable change in the level of mRNA compared to other RNAs. Such a change should be revealed as a color bias in the scatter plots. Examination of scatter plots with RNA from polio infected cells, using data prior to normalization, did not reveal any color bias beyond what was observed for arrays based on cells given Ifn or vaccinia virus. Thus poliovirus did not cause any drastic selective reduction of mRNA at the time of sampling.

As to changes in gene expression, Ifn had a more pronounced effect than did poliovirus, witnessed both by the number of genes affected (Table 1), and the degree of changes in the affected genes (data not shown).

### Comparison of viruses and interferon

One purpose of the current experiments was to find candidate genes that might be involved in a cellular response to virus infections. Thus the lists of up- or down-regulated genes were compared to see to what extent the various lists comprised the same genes (Table 2). Only 3 genes were up-regulated by both poliovirus and vaccinia virus (whether comparing the 8 h or the 16 h poliovirus lists), and none of the down-regulated genes were shared. It was expected that the cells would deal with these rather dissimilar viruses by different responses. More surprisingly, when comparing the effects of poliovirus at 8 and 16 h, there were only four common genes. The p-value for this observation to be random, assuming a Poisson distribution, is approximately 0.06, implying that even a random draw of genes could come up with four shared genes.

**Table 2:** Genes shared by pairs of the up- or down-regulated gene lists presented in Table 1.

Comparisons	Shared up-regulated genes	Shared down-regulated genes
Ifn-8 AND Ifn-16	143	8
Pol+Ifn-8 AND Pol+Ifn-16	131	13
Pol-8 AND Ifn-8	82	0
Pol-8 AND Pol+Ifn-8	57	0
Pol-16 AND Ifn-16	19	9
Pol-16 AND Pol+Ifn-16	46	20
Pol-8 AND Pol-16	4 (MATN4, UPP2, WWTR1, H300004086)	0
Vac-4 AND Pol-8	3 (CXXC5, MATN4, MPP4)	0
Vac-4 AND Ifn-8	3 (CXXC5, IER2, ZFP57)	0

The above observation might question the validity of the data. However, when comparing the effect of poliovirus to that of Ifn, considerable fractions of the genes were shared, particularly at 8 h. This was the case whether examining results from cells exposed only to Ifn at 8 h or 16 h, or to both virus and Ifn. Vaccinia virus, on the other hand, did not induce the same changes as Ifn.

The present cells (embryonic fibroblasts) would be expected to respond to, but not necessarily produce, interferon-α. Fibroblasts are known to produce interferon-β more readily. Although some interferon transcripts (IFNA14, IFNG and the related IL6), were detected at low levels in some hybridizations, none of the viruses induced to any measurable extent any interferon genes. Interferon-β (IFNB1) was included in the arrays (H300002697), but with signals consistently at or below background. Thus the similarity between the genes induced by poliovirus and interferon-α did not appear to be due to viral induction of interferons. At least three interferon receptors were clearly expressed (IFNAR2, IFNGR1 and IFNGR2), but none of them were induced by either virus or Ifn.

The observation that poliovirus and Ifn induced many of the same genes suggests that these genes may be involved in viral defense. The microarray data may not be sufficiently sensitive to exclude some induction of interferon genes due to poliovirus infection. Yet it is conceivable that the putative defense genes (the genes up-regulated by both Ifn treatment and poliovirus infection) were induced in an interferon independent way.

The lists of up-regulated genes were further examined in order to suggest candidate defense genes (see below).

### Gene ontology analyses

Gene ontology (GO) analyses were performed in order to characterize the cellular response, and as part of the search for candidate defense genes. It was focused on to what extent particular Biological process categories would be over-represented on the lists of genes up-regulated by the two viruses.

In the case of vaccinia virus, the most selectively (based on p-values) up-regulated GO categories were Cell proliferation and Wound healing (Table 3). A positive regulation of DNA replication/cell proliferation would be expected for DNA viruses that replicate in the nucleus, a contention supported by reports on other DNA viruses [17,18]. Pox viruses, however, replicate in the cytoplasm, and should thus be less dependent on the DNA replication machinery of the host. A previous study on vaccinia virus reported a down-regulation of genes involved in cellular replication [7]. In the present analyses the term Cell proliferation covers a broader range of genes than what is included in "cellular replication". Moreover, some of the genes on the lists may actually function as down-regulators of replication.

**Table 3:** Gene ontology analyses of up-regulated genes.

Gene lists used for comparison	Biological process category	Number of overrepresented genes/all genes in category (p-value)
Vac-4 up-regulated	Regulation of cell proliferation GO:0042127	7/181 (0.000)
	Positive regulation of cell proliferation GO:0008284	5/80 (0.000)
	Wound healing GO:0042060	4/57 (0.001)
Pol-8 up-regulated	Antigen processing (GO:0030333)	3/39 (0.010)
	Antigen presentation (GO:0019882)	3/46 (0.016)
	Regulation of signal transduction (GO:0009966)	6/175 (0.016)
	Regulation of I-kappaB kinase/NF-kappaB cascade (GO:0043122)	4/85 (0.017)
	Response to extracellular stimulus (GO:0009991)	2/18 (0.018)
Pol-16 up-regulated	DNA replication (GO:0006260)	9/171 (0.000)
	Response to wounding (GO: 0009611)	8/213 (0.003)
	Regulation of signal transduction (GO:0009966)	7/175 (0.004)
	Response to stress (GO:0006950)	17/758 (0.007)
	Regulation of MAPK activity (GO:0043405)	3/37 (0.009)
	RNA modification (GO:009451)	2/16 (0.0015)
	Cell proliferation (GO:0008283)	11/433 (0.017)

The cells were incubated with poliovirus (Pol) for 8 or 16 h, or with vaccinia virus (Vac) for 4 h prior to microarray examination. Lists of up-regulated genes were compared with the lists of all the genes expressed in that experiment, using the Target-master statistical test available under GeneTools [37]. Only the more significant and more relevant results are presented. The total number of annotated, expressed genes was 10195.

While stimulation of cell proliferation is unlikely to reflect cellular defense, the four up-regulated genes involved in Wound healing could possibly offer a clue in this direction. However, closer inspection of the genes, as to known functions, as well as degree and consistency of up-regulation, did not warrant listing them as putative antiviral defense genes.

Poliovirus also selectively up-regulated genes involved in Cell proliferation and DNA replication at 16 h, but in this case a closer examination suggested that the genes in question were more likely to inhibit these processes. I.e., they could be part of the closedown of the cell, and as such potentially a response to limit viral replication. Genes involved in the Regulation of signal transduction were up-regulated at both 8 and 16 h. Moreover, genes involved in Response to extracellular stimulus and Antigen processing/presentation were selectively up-regulated at 8 h, while Inactivation of MAPK activity, RNA modification and Response to wounding/stress were up at 16 h. The genes included in these lists might offer clues to potential defense mechanisms, and were singled out for further examination.

GO categories such as Response to virus, Regulation of antiviral response, and Cellular defense response were notoriously absent in the lists of selectively up-regulated Biological processes. Yet, the strong correlation between genes induced by poliovirus and those induced by Ifn alone, combined with the observation that Ifn did inhibit poliovirus replication, suggest a distinct antiviral response. A possible explanation for the apparent discrepancy is that the GO analyses fail due to incomplete annotations. In other words, many of the genes directly or indirectly involved in, e.g., Response to virus, lack annotations reflecting this function. Although the response of the cells to limit viral replication may be mediated by genes whose primary function is not antiviral defense, the observation that so many genes were induced by both Ifn and poliovirus suggests that at least some of these genes serve as antiviral response genes in a more general way. Of all the expressed genes, 39 had the annotation Response to virus. Most of these genes appeared to have other main functions, and their association with viral response may not be well documented. Moreover, most of them were neither up- or down-regulated in the present experiments.

Programmed cell death, e.g. apoptosis, is a presumed host defense response in the case of poliovirus infection (reviewed in [19]). In the present experiments, however, there were no significant changes in the genes involved in this processes after either 8 h or 16 h of poliovirus infection. There was, however, a tendency for the up-regulation of Negative regulation of apoptosis genes (p = 0.06) at 16 h (but not at 8 h), suggesting that the virus was active in postponing cell death.

GO analyses were considered valuable as they offered hints as to the gross nature of cellular changes. For pinpointing antiviral defense genes, however, they may be of limited use.

### Candidate genes for viral defense

Two lists of putative antiviral defense genes were created, one containing genes with established annotations (Table 4), the other with genes with little or no functional information (see below). Even in the case of the annotated genes, functional information was in most cases uncertain and limited. The main criteria for inclusion in either list were as follows: 1, being up- (or down-) regulated by both poliovirus and Inf; 2, being appreciably up- (or down-) regulated, preferably to high expression values and with good concordance between parallels; 3, being up- (or down-) regulated by both viruses; and 4, having annotations or literature references suggestive of an antiviral role.

**Table 4:** Annotated candidate genes for viral defense listed alphabetically.

Symbol	Name	Indication	Expression level	Induction	Comments
CXXC5	CXXC finger 5	Up in Pol-8, Vac-4, Ifn-8 and BKV	H	H	DNA binding/regulator of gene transcription
HMGB2	High-mobility group box 2	Induced by both Pol and Ifn (16 h)	H	M	DNA binding and regulation, cytokine
ITGAV	Integrin α5	Down or border line down in all experiments	M-H	L (or just below L)	Membrane protein associated with adhesion and signal transduction, virus uptake
LY6E	Lymphocyte antigen 6 complex locus E	Induced by both Pol and Ifn (8 h)	M-H	M-H	Cell surface receptor involved in defense
LZTR1	Leucine-zipper-like transcription regulator 1	Induced by both Pol and Ifn (8 h)	H	H	Transcription regulator
MATN4	Matrilin4	Up in Pol-8, Pol-16, Vac-4, Ifn-16 and BKV	M-H	M-H	Extracellular matrix adapter?
MT1L	Metallothionein 2A	Induced by both Pol and Ifn (16 h)	H	M	Metal binding/homeostasis
PKM2	Pyruvate kinase, muscle	Induced by both Pol and Ifn (8 h)	H	H	Kinase activity
RND3	Ras homolog gene family E	Induced by both Pol and Ifn (16 h)	H	M	Actin cytoskeleton
SIGLEC1	Sialoadhesin	Induced by both Pol and Ifn (8 h)	H	H	Immunoglobulin adhesion molecule on cell surface
UBC	Ubiquitin C	Induced by both Pol and Ifn (8 h)	H	H	Selective protein degradation

Expression level: below 500 is low, 500–1500 medium, above 1500 high. Induction level: /log2/ between 0.6–0.9 is low, 0.9–1.2 medium, >1.2 high.

One gene, MATN4 (matrilin4) was induced in all three virus experiments. The odds for this observation to be random is p = 0.01. It was also induced by Ifn and Pol+Ifn at 16 h, but not at 8 h. Moreover, the same gene was induced in a different cell type by the polyomavirus BKV [20]. Collectively these observations are highly unlikely to be random, and consequently suggest a role in antiviral defense. Matrilins are presumed to be involved in the formation or function of extracellular matrixes [21], but the specific function of MATN4 is unknown. In the experiments with Coxsackie B3 and mouse myocarditis, other extracellular matrix genes were induced [6].

The only other gene to be induced by poliovirus (but only at 8 h), vaccinia virus and BKV was CXXC5. It belongs to a family of proteins with a CXXC motif within their DNA-binding domain, which presumably are involved in the regulation of transcription. As such one might envision a role in viral defense.

Only one of the genes with decreased transcription was considered of particular interest. ITGAV, an integrin alpha V chain gene associated with adhesion and signal transduction, was down or borderline down in all the present experiments, including those with Ifn alone. The alpha V integrins recognize a variety of ligands for signaling, and are involved in cell migration, adhesion and proliferation. They have also been implicated in the binding and uptake of enteroviruses [22].

The remaining genes in Table 4 were up-regulated independently by poliovirus and Ifn: HMGB2 is closely related to HMGB1, which is induced as a consequence of infection by several viruses [23]. Besides being presumed to be involved in transcriptional regulation, it appears to be released as a proinflammatory cytokine. LY6E is annotated as being involved in cellular defense, and has been implicated in viral resistance in chicken [24]. LZTR1 is a transcription regulator. MT1L belongs to the metallothionein family which regulates distribution of trace metals. Metallothioneins have been shown to be up-regulated due to various viral infections, including Coxsackievirus [25]. PKM2 has presumably its primary role in glycolysis, but pyruvate kinases have also been associated with viral defense reaction in plants [26], and in the induction of human cellular proliferation by viral oncoproteins [27]. RND3 was one of several (including FSCN1 and CKAP4) up-regulated cytoskeleton related genes, suggesting that the cytoskeleton, and possibly membrane transport, are an issue. RND3 is associated particularly with actin filaments. SIGLEC1 was one of the most induced genes by poliovirus at 8 h. The gene has been implicated in the attempt of the related human rhinovirus to subdue host response [28], but the present observation that it was induced by both poliovirus and Ifn suggests a role in fighting poliovirus. UBC is involved in selective protein degradation, a function that may serve either virus or cell [29].

In addition to these genes, several HLA related genes were induced by poliovirus, four of them also by Ifn: HLA-A, HLA-B, H300022822 (HLA-A related), and H300015425 (HLA-G histocompatability antigen). Their presumed relevance in the present context is in antigen presenting. They are not included in Table 4.

A number of genes with no official annotations were also considered as candidates for viral defense, based on criteria 1–3 above: H300007844 (a seven transmembrane helix receptor candidate), H300007235, H300010054, H300002629, H300008732 (possibly related to SHP-1 in mouse, which inhibit replication of murine encephalomyelitis virus [30]), H300004369, H300016649 (apparently a fibronectin related gene, fibronectin seems to be imported for replication by various viruses [31]), H300009394, H300007545, H300005013, H200010659 and H300006939 (C14orf59, containing a zinc finger domain, the gene most highly induced by poliovirus, but with no known function).

## Conclusion

Under the present conditions, both poliovirus and vaccinia virus were able to exploit the cells to the extent that the cells eventually died. The cells, however, may still have launched a defense. In the case of poliovirus, a defense is suggested by the observation that the virus alone induced a considerable fraction of the genes induced by Ifn (at a concentration of Ifn that substantially reduced viral replication).

The original assumption was that the same viral defense genes might be involved in the defense of different viruses, while genes induced to promote a particular virus would more likely be specific for that virus. Somewhat surprisingly, the cellular response was widely different, not only depending on type of virus, but also on the stage of viral life-cycle. However, the considerable number of genes induced by both poliovirus and Ifn, suggested that this virus was fought by "general response" genes, i.e., interferon induced genes. Moreover, it is possible that interferon itself is not required for the induction of these genes. The suggested list of candidate antiviral defense genes was based to a large extent on the comparison between poliovirus and Ifn induced genes.

In order to understand the interaction of virus and host, it is important to examine cells that are reasonably synchronously infected. Even within the relatively short time-frame from 8 to 16 h, the response to poliovirus changed beyond recognition. The observation highlights the problem of comparing results from different laboratories, where many factors are different, including cells, viral strains and time points used. Not surprisingly, most of the genes induced in the present experiments were not on the lists of up-regulated genes even in previous reports on related viruses. Apparently there is not much of a standardized response, but rather a question of adapting the response to the cell type and the situation at hand.

The other report using microarrays to analyze cellular transcripts upon poliovirus infection looked specifically at polysome-associated mRNAs in HeLa cells [1]. Cytoplasmic actin (ACTB) was used as a control for mRNA expression. Their results corresponded to the present ones in that the ACTB gene was highly expressed in both viruses infected cells and control cells. However, two other genes that were clearly up-regulated in the previous publication (MYC and MAP2K3), were expressed, but either unchanged or only slightly increased by virus in the present results.

One should bear in mind that non-coding genes are not covered by the present arrays, and that these too may be involved in viral defense, particularly through RNA silencing [32,33]. However, most miRNAs and siRNAs are presumably involved in the regulation of the expression of protein coding genes [34]. Thus changes in their activity are likely to be reflected in changes of mRNA levels measurable using the present microarrays, with the possible exception of siRNAs directed specifically at the viral genes.

## Methods

### Cells, viruses and RNA preparation

A human embryonic fibroblast-like (HE) cell line was used, originally obtained from an 8 week old fetus. The fetus was treated with trypsin, and the cell suspension seeded out and passaged several times until the remaining cells had a uniform fibroblast appearance. For the present purpose the cells were grown in 175 cm^2 ^Nunc bottles with Dulbecco's Modified Eagle Medium (MEM; Gibco, Invitrogen Corporation, USA) and 10% fetal bovine serum (Gibco) between passages 16 and 18. The experiments were done on cells in growth phase, that is, the cells were split 24 h prior to the start of the experiments, and seeded out to a density such that the flasks would not have reached confluence by the time of harvest. Infection was performed by removing the medium, adding virus or mock (conditioned medium without virus), and incubating the cells on a tilting platform for 1 h at room temperature. The inoculum was removed after 1 h in order to obtain a more synchronous replication. The cells were subsequently washed once with PBS, and the original medium was reintroduced. The harvesting (of approximately 10^7 ^cells) was performed by removing the medium, washing once with PBS, exposure to 4 ml of trypsin for 2 min at 37°C, inactivation of trypsin with a few drops of fetal bovine serum, transfer to tubes, and 2 min centrifugation at 2000 rpm in a Microfuge (Eppendorf, USA). The pellets were immediately resuspended in 500 μl lysis buffer from the extraction kit, frozen, and stored at -70°C. Parallel bottles of cells were retained to estimate the percentage of infected cells, to determine at what time the viruses would cause cytopathology, and for assessing the viability of non-infected cells.

Total RNA was extracted from virus- or mock-infected cells with the GenElute Mammalian Total RNA Kit (Sigma, MO, USA). The concentration and quality of RNA was determined by UV spectrophotometer measurements using a Nanodrop (ND-1000, Nanodrop Technologies, Rockford, USA), and the lack of degradation controlled for by gel-electrophoresis, using an Agilent 2100 Bioanalyzer (Agilent Technologies, CA, USA) according to manufacturers' recommendations. The yield of RNA was between 32 and 118 μg per bottle of cells.

The type 1 poliovirus was the Sabin strain obtained from ATCC (VR-1562). The vaccinia virus was the one previously used as live vaccine in Norway. The vaccinia virus was adapted to HE cells by passaging 10 times. The titers of the viral stock solutions were determined by endpoint dilution on the same cell line.

In the case of poliovirus, the virus was added at a multiplicity of infection (MOI) of 10, except for experiments with Ifn (Interferon α-IFN-Le, Sigma I 2396), where it was added at 2 MOI in order to ensure an obvious effect of the cytokine. Ifn was added 24 h prior to the virus at a concentration of 200 U/ml, and then added again after the inoculation. At a MOI of two, cytopathogenic effect was observed after 2–3 days in the absence of Ifn and after 6–7 days with Ifn. At 10 MOI cytopathogenicity started within 2 days. In both cases 70 – 90% of the cells would display cytopathogenicity within 3 days. The difference in MOI did not drastically change the effect of the virus.

In the case of vaccinia virus, the virus was added at 2 MOI. A rabbit anti-vaccinia serum was produced by inoculating animals with the virus. Using this serum it was found that 60–70% of the cells produced vaccinia antigens within 10 h of inoculation.

Three parallel cell flasks were included for each type of treatment, each time point and for controls (mock infected cells). For each experiment, the cells were seeded out from one population of cells, and the various bottles were handled in the same way. The RNA from the three control parallels were mixed prior to labeling, while in the case of the virus infected cells, RNAs from the three independent flasks were labeled separately and used for different microarrays. The rational for mixing the control RNAs was to obtain a more uniform baseline.

### Microarrays

RNA was converted to fluorescence-labeled cDNA with the FairPlay™ aminoallyl kit, using 20 μg of total RNA per labeling, and Cy5 or Cy3 as fluorescent dyes (Amersham International, UK), according to manufacturer's recommendations. All the presented data were obtained by hybridizations utilizing 35 k human 70-mer oligonucleotide-based microarrays provided by the Norwegian Microarray Consortium [35]. The microarrays were spotted on Corning UltraGAPS glass-slides, using the Operon Human Genome Oligo Set Version 3.0, with 34580, 70-mer probes representing 24 650 known or putative genes and 37 123 gene transcripts (Operon Biotechnologies, Huntsville, AL, USA). Controls, in the form of Lucidea Microarray ScoreCard v1.1 (Amersham Biosciences), were included. Automated hybridization was performed for 12 h at 45°C with the ChipMap80 kit (including the ChipHybe80 buffer) on the Ventana Discovery system (Ventana Medical Systems, Tucson, AZ, USA). The arrays were scanned on an Agilent fluorescence scanner (Axon Instruments, Foster City, CA, USA) with pixel size of 10 μm.

An initial set of experiments were performed with smaller cDNA arrays (also provided by the Norwegian Microarray Consortium), which consisted of 15 000 spots with IMAGE cDNA clones from Research Genetics (AL, USA). In-house produced PCR products reflecting poliovirus polyA-RNA (541 nt products from the 3'end of poliovirus types 1, 2 and 3) were included in these arrays. In this case, the labeling of the mRNA was similar, but the hybridization and washing was performed in a GenTAC Hyb-Station (Genomic Solutions, USA) using a standard protocol [35] and 20 μg of each RNA. Data from these arrays were used only as controls to validate results obtained from the oligo-based arrays. Comparisons revealed a general concordance as to the clearly up- or down-regulated genes. The agreement decreased when looking at low-expressed and marginally up- or down-regulated genes.

### Microarray analyses

Quality control of the scanned images (tiff-files) and conversion to numerical values were performed using the GenePix Pro 4.1 (Axon Instruments). The gpr-files obtained were further analyzed using J-Express pro2.7 [36]. Mean FG intensities were used as input values. Spots with less than 60% of the pixels (in either Cy5 or Cy3) below background + 2 SD were removed. Controls as well as flagged or empty spots were also filtered away before Global Lowess Normalization. Further quality control was performed using the various Plot and Spot Image View options in J-Express. Reporters for which at least two of the three parallels complied with the above filtering were considered expressed, and the relevant data transferred to Excel. The average ratios, as well as average red and green intensities, for the three parallels were calculated. Reporters with average red/green log_2 _ratios above 0.6 or below -0.6 (adjusted for color bias) were considered respectively up- or down-regulated. Subsequent gene ontology (GO) analyses were based on these lists. The numbers of up- and down-regulated genes are listed in Table 1.

The gene ontology program eGOn V2.0 and the NMC Annotation Database V2.0, both available at GeneTools [37], were used to examine gene function, based on gene annotations available as of June 2006. To compare up- or down-regulated genes with all expressed genes the "Target-Master test" was used. The following internet-based resources gave additional information as to gene functions: PubGene [38], Operon's OMAD [39], Ensembl-human [40], Panther [41], and GeneCards [42]. HUGO GeneSymbols are preferentially used. The complete microarray data are available at the Gene Expression Omnibus [43] with the accession no GSE5549.

## Competing interests

The author(s) declare that they have no competing interests.

## Authors' contributions

MG cultured the poliovirus and carried out the molecular biology, i.e., preparing samples for microarray and participated in the laboratory part of the microarray work. GH cultured the vaccinia virus and provided support for the culturing of poliovirus. BG designed the study, was involved in the microarray laboratory work and did the data analyses.
